# Increased Succinate Accumulation Induces ROS Generation in *In Vivo* Ischemia/Reperfusion-Affected Rat Kidney Mitochondria

**DOI:** 10.1155/2020/8855585

**Published:** 2020-10-13

**Authors:** Justina Kamarauskaite, Rasa Baniene, Darius Trumbeckas, Arvydas Strazdauskas, Sonata Trumbeckaite

**Affiliations:** ^1^Neuroscience Institute, Lithuanian University of Health Sciences, Eivenių g. 4, LT-50009 Kaunas, Lithuania; ^2^Department of Pharmacognosy, Medical Academy, Lithuanian University of Health Sciences, Sukileliu pr. 13, LT-50009 Kaunas, Lithuania; ^3^Department of Biochemistry, Medical Academy, Lithuanian University of Health Sciences, Eivenių g. 4, LT-50009 Kaunas, Lithuania; ^4^Department of Urology, Medical Academy, Lithuanian University of Health Sciences, Eivenių g. 2, LT-50009 Kaunas, Lithuania

## Abstract

Mitochondria are recognized as main reactive oxygen species (ROS) producers, involving ROS generation by mitochondrial complexes I and III. Lately, the focus has been shifting to the ROS generation by complex II. Contribution of complex II (SDH) to ROS generation still remains debatable, especially in *in vivo* settings. Moreover, it is not completely defined at what time of ischemia the first alterations in mitochondria and the cell begin, which is especially important with renal arterial clamping *in vivo* during kidney surgery, as it predicts the postischemic kidney function. The aim of this study on an *in vivo* rat kidney ischemia/reperfusion model was to determine if there is a connection among (a) duration of kidney ischemia and mitochondrial dysfunction and (b) succinate dehydrogenase activity, succinate accumulation, and ROS generation in mitochondria at low and saturating succinate concentrations. Our results point out that (1) mitochondrial disturbances can occur even after 30 min of kidney ischemia/reperfusion *in vivo* and increase progressively with the prolonged time of ischemia; (2) accumulation of succinate in cytosol after ischemia/reperfusion correlated with increased H_2_O_2_ generation mediated by complex II, which was most noticeable with physiological succinate concentrations; and (3) ischemia/reperfusion induced cell necrosis, indicated by the changes in LDH activity. In conclusion, our new findings on the accumulation of succinate in cytosol and changes in SDH activity during kidney ischemia/reperfusion may be important for energy production after reperfusion, when complex I activity is suppressed. On the other hand, an increased activity of succinate dehydrogenase is associated with the increased ROS generation, especially with physiological succinate concentrations. All these observations play an important role in understanding the mechanisms which occur in the early phase of ischemia/reperfusion injury *in vivo* and may provide new ideas for novel therapeutic approaches or injury prevention; therefore, more detailed studies are necessary in the future.

## 1. Background

Ischemia/reperfusion (I/R) injury of the kidney is a complex process; it involves free radical overproduction, inflammation, disturbances in microcirculation, and damage of mitochondria, leading to postischemic kidney dysfunction [1]. Oxidative stress plays a central role in this process [2]. Mitochondria in the cell supply the energy in the form of ATP for basal cell functions as well as for cellular repair and regeneration; therefore, there is an emerging evidence about the importance of these organelles in I/R injury [3]. In previous studies, it has already been shown that I/R leads to the reduced activity of mitochondrial respiratory chain complexes, ATP depletion, calcium accumulation, mitochondrial permeability transition pore opening, and an increase in reactive oxygen species (ROS) production; however, the complete mechanisms and the interaction between these processes are not fully elucidated. Moreover, it is not clear at what time of ischemia the first alterations in mitochondria and the cell begin, what duration of ischemia is already critical for the mitochondria, and how mitochondrial impairment progresses during ischemia. This is extremely important during renal surgery, when arteria is clamped for a certain period, as it determines the postischemic kidney recovery processes.

It is generally accepted that mitochondrial ROS generation is a potential contributory factor for kidney I/R injury and that ROS generation can be exacerbated during the postischemic reperfusion stage. Mitochondrial ROS not only drive acute damage but also initiate the pathology that develops over the minutes, days, and weeks following reperfusion. The mitochondrial respiratory chain complexes, namely, I and III, were considered as the main ROS producers under normal and pathological conditions [4, 5], while the contribution of complex II (SDH) was considered to be negligible [6]. However, in recent years, the impact of mitochondrial complex II in the generation of ROS has been getting more and more scientific interest. Chouchani et al. observed that ischemia in various organs, such as the brain, heart, liver, and kidney, induces accumulation of Krebs cycle intermediate succinate in mitochondria which was reoxidized by succinate dehydrogenase at reperfusion [7]. The authors proposed an interesting fact that the succinate-driven reverse electron transport through complex I was the main source of ROS under these conditions [7]. Quinlan et al., investigating skeletal muscle mitochondria, suggested that mitochondrial complex II generated superoxide radical in both, the forward and reverse reactions, and the authors also concluded that complex II could be a relevant producer of ROS under *in vivo* conditions such as hypoxia [8]. As far as we know, there are no data discussing whether kidney ischemia/reperfusion injury correlates with succinate accumulation and succinate dehydrogenase-induced ROS generation *in vivo* and whether it depends on the duration of ischemia *in vivo*.

The aim of this study on the *in vivo* rat kidney ischemia/reperfusion model was to determine if there is a connection among (a) duration of kidney ischemia and mitochondrial dysfunction and (b) succinate dehydrogenase activity, succinate accumulation, and ROS generation in mitochondria at low and saturating succinate concentrations.

## 2. Materials and Methods

### 2.1. Animals and Experimental Ischemia/Reperfusion *In Vivo* Model

The experimental procedures were performed according to the permission of the Lithuanian Committee of Good Laboratory Animal Use Practice (No. 0217/2011). Male Wistar rats (2-4 month-old), weighing 200-250 g, were housed under standard laboratory conditions, maintained on natural light and dark cycle and had free access to food and water. On the operation day, rats were anesthetized with a combination of an intraperitoneal injection of pentobarbital and ketamine. Pentobarbital (200 mg/mL) was diluted with isotonic NaCl solution (0.9%) to a concentration of 20%, and an intraperitoneal injection of 0.1 mL/100 g rat weight was given. Ketamine (100 mg/kg) was administered intramuscularly (0.1 mL/100 g rat weight). Body temperature was kept constant at 37°C with a warming pad. A Yasargil clip (Aesculap, Tübingen, Germany) was placed over a rat's renal artery to induce renal normothermic ischemia (37°C) for 20, 30, 40, and 60 min with subsequent 30 minutes of reperfusion after removing the clip.

### 2.2. Preparation of Isolated Kidney Mitochondria

At the end of ischemia/reperfusion, the kidney was removed and placed into ice-cold medium containing 10 mM Tris·Cl, 250 mM sucrose, and 1 mM EDTA (pH 7.3); the tissues were cut and homogenized in a glass-teflon homogenizer as described in [9]. The protein concentration was determined by the biuret method as described in [9]. Supernatants were collected and used for the determination of lactate dehydrogenase activities.

### 2.3. Measurement of Kidney Mitochondrial Respiration

**Figure 1:**
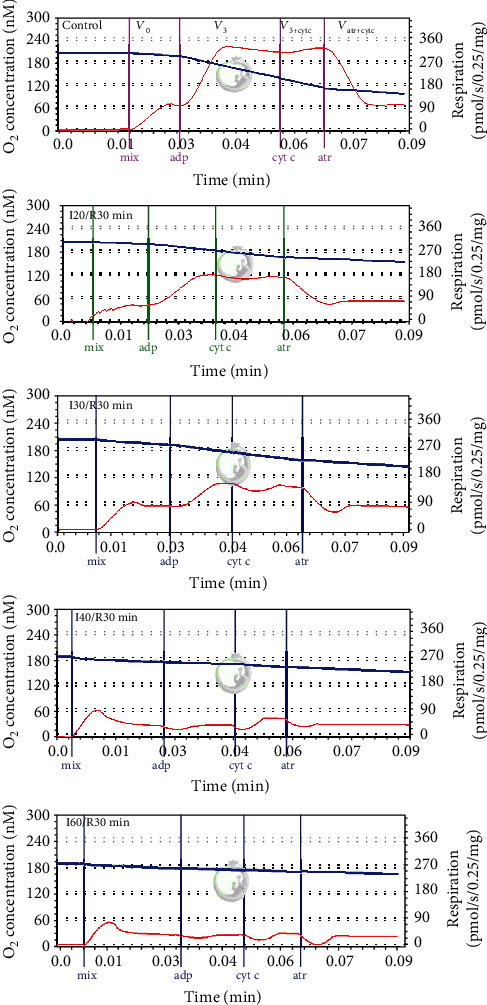
Original curves of kidney mitochondrial respiration in the control and ischemia/reperfusion (I/R) groups. Mitochondrial respiration rate was measured as described in Methods using 5 mM glutamate plus 5 mM malate as substrates. The blue trace represents oxygen concentration (nmol/mL), and the red trace represents oxygen flux. I20/R30, I30/R30, I40/R30, and I60/R30 refer to different ischemia durations (respectively, 20, 30, 40, and 60 minutes) following by 30-minute reperfusion. *V*_0_: routine respiration rate in the presence of 0.5 mg/mL of mitochondria and substrates; *V*_3_: state 3 respiration rate in the presence of 1 mM ADP; *V*_3+cyt c_: state 3 respiration rate in the presence of 32 *μ*M cytochrome c; *V*_ATR (+cyt c)_: respiration rate in the presence of 0.12 mM atractyloside and 32 *μ*M cytochrome c.

Mitochondrial oxygen consumption rates was measured using Oroboros oxygraph-2 k in 2 mL incubation medium containing 10 mM Tris·Cl, 5 mM KH_2_PO4, 150 mM KCl, 1 mM MgCl_2_ × 6H_2_O, pH 7.2, 37°C with 5 mM glutamate + 5 mM malate, or 15 mM succinate + 2 mM amytal (an inhibitor of complex I) as respiratory substrates. All experimental conditions are described in [10]. The mitochondrial routine respiration rate (*V*_0_) was recorded in the medium supplemented with mitochondria and substrates (complex I-dependent substrate: glutamate 5 mM + malate 5 mM, or complex II-dependent substrate: succinate 15 mM + 2 mM amytal as complex I inhibitor); original curves of mitochondrial respiration are seen in Figure 1. The effect of cytochrome c on respiration rate (indicating mitochondrial outer membrane permeability) was determined by adding 32 *μ*M cytochrome c (*V*_3+cyt c_) to mitochondria respiring in state 3. Then, the respiration was inhibited with 0.12 mM atractyloside, an inhibitor of ADP/ATP translocator (*V*_ATR_). Changes in atractyloside-inhibited respiration rates and routine respiration rates indicated the changes in the permeability of mitochondrial inner membrane. The respiratory control index (RCI) for glutamate/malate was calculated as the ratio of respiration rates *V*_ADP_/*V*_0_ and for succinate as the ratio *V*_ADP_/*V*_ATR_. Cytochrome c effect was calculated as the ratio V_ADP+cyt c_/V_ADP_. Datlab 5 software (Oroboros Instruments) was used for real-time data acquisition and data analysis. Oxygen consumption rates were expressed as pmol/s/0.25 mg mitochondrial protein.

### 2.4. Measurement of Mitochondrial Respiratory Chain Complexes I and II+III and Succinate Dehydrogenase Activity

The activity of complex I was measured spectrophotometrically at 340 nm, according to the kinetics of NADH oxidation [9]. The activity of complex II+III in mitochondria was measured following the reduction of cytochrome c at 550 nm (after the addition of succinate, respectively) using an extinction coefficient for cytochrome c of 21.1/mM/cm as described in [11]. Succinate dehydrogenase activity was measured at 600 nm following the reduction of 2,6-dichlorophenolindophenol using an extinction coefficient of 19.1/mM/cm as described in [12].

### 2.5. Measurement of Succinate Amount in Cytosolic and Mitochondrial Fractions

Lyophilized kidney cytosolic and mitochondrial fractions were used for chemical analysis. The samples (10 mg) were dissolved in tetrahydrofuran (≥99.9% (HPLC) Sigma-Aldrich, Germany), transferred to a thermostatic ultrasound bath (Heidolph, Schwabach, Germany), and extracted for 15 minutes. The extracts were centrifuged for 5 min at 1699.36 × g (fixed angle rotor BRK5412, rotor type 12 × 15 mL). The supernatants obtained were removed from sediment, filtered through a Q-Max membrane filter (25 mm in diameter, 0.45 *μ*m in pairs; Frisenette, Knebel, Denmark), and used for chromatographic analysis as indicated in [13].

### 2.6. Measurement of H_2_O_2_ Generation in Kidney Mitochondria

The generation of H_2_O_2_ in kidney mitochondria was determined fluorimetrically (fluorometer, Thermo Scientific) as described in [9]. Respiratory chain complex inhibitors: complex I inhibitor rotenone (5 *μ*M) and complex III inhibitor myxothiazol (2 *μ*M), were added to the appropriate wells. Fluorescence signal was calibrated using known amounts of H_2_O_2_.

### 2.7. Measurement of Lactate Dehydrogenase Activity

Lactate dehydrogenase (LDH) activity in cytosolic fractions was measured spectrophotometrically by monitoring NADH oxidation rate at 340 nm as described in [9].

### 2.8. Statistical Analysis

Data are presented as the mean ± SEM of 4-11 separate experiments. The mean for individual experiment was obtained from at least three repetitive measurements. Statistical analysis was performed using the software package SPSS version 16.0 for Windows. *p* < 0.05 was considered the level of significance.

## 3. Results

### 3.1. Changes in Kidney Mitochondrial Oxidative Respiration Rates after Postischemic Reperfusion

One of the consequences of ischemia/reperfusion induced injury is the suppression of mitochondrial respiration rates and activities of respiratory chain complexes. Our previous studies have revealed that kidney ischemia *in vitro* causes suppression of mitochondrial function in an ischemia-duration dependent manner [14]. Whether *in vitro* results could be extrapolated to *in vivo* experiments, the question remains open. Therefore, in this study, mitochondrial oxidative phosphorylation rates with two different substrates, complex I-dependent glutamate/malate and complex II-dependent succinate, first of all were tested in the *in vivo* kidney ischemia/reperfusion model.

Figure 1 shows original mitochondrial respiratory curves in control and ischemia/reperfusion-affected mitochondria. Summary of results are presented in Table 1. Obvious changes were found in our study already after 30 min ischemia/reperfusion; i.e., mitochondrial state 3 respiration rate (in the presence of external ADP) was suppressed by 37% (*p* < 0.05) as compared to control (i.e., nonischemic) mitochondria (Table 1). After ischemia was prolonged to 40 min and 60 min, a further decrease was observed in state 3 respiration rate (by 76% and 86%, respectively) and in atractyloside-inhibited respiration rate *V*_ATR_ (by 57% and 65%, respectively), which shows changes in permeability of the inner mitochondrial membrane. The routine respiration (*V*_0_) rate decreased by 54% and 49%, respectively (*p* < 0.05) (Table 1). As a consequence, the respiratory control index (RCI) also diminished by 30%, 51%, and 73% (*p* < 0.05) during longer ischemia/reperfusion periods (30 min, 40 min, and 60 min) (Table 1). For instance, the RCI with glutamate/malate as substrates after 60 min ischemia/reperfusion was found to be 0.89, indicating full mitochondrial dysfunction.

In contrast, mitochondrial state 3 respiration with succinate (Table 2) as a substrate was drastically affected (decreased by 80%) only after a longer period (60 min) of ischemia/reperfusion, whereas during ischemia periods of 20 min, 30 min, and 40 min, it even caused a slight increase (of 29%, 41% (*p* < 0.05), and 14%, respectively) in state 3 respiration rates. The routine respiration rate (*V*_0_) as well as *V*_ATR_ did not change statistically significantly after 20 min and 30 min of ischemia/reperfusion and increased by 71% and 97% after 40 min of ischemia/reperfusion (*p* < 0.05, Table 2) causing a 41% decrease in RCI (*p* < 0.05, Table 2). As can be seen, when the ischemia period was prolonged to 60 min, the mitochondrial function was fully disturbed—the RCI was found to be 1.05, and there was no coupling between oxidation and phosphorylation. Additionally, we also checked the effect of ischemia/reperfusion on the intactness of mitochondrial outer membrane (after addition of exogenous cytochrome c in state 3). The addition of cytochrome c had none or a slight effect on the intactness of mitochondrial outer membrane after a short (20 min and 30 min) ischemia/reperfusion period, whereas after 40 min and 60 min of ischemia/reperfusion, it stimulated the state 3 respiration rate with both substrates, glutamate/malate, and succinate, but with succinate, it was much more obvious (respiration rate increased two and four times, respectively, *p* < 0.05, Tables 1 and 2).

### 3.2. Kidney Ischemia/Reperfusion Affects the Complex I, Complex II+III, and Succinate Dehydrogenase Activity

**Figure 2:**
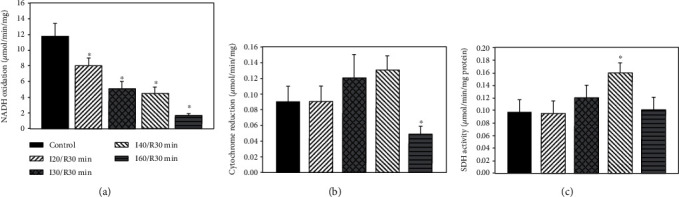
Effect of ischemia on complex I (a), complex II+III (b), and succinate dehydrogenase (c) activity in kidney mitochondria. The complex I activity was measured spectrophotometrically at 340 nm as described in Methods. ^∗^*p* < 0.05*vs.* control. The complex II activity was measured spectrophotometrically at 550 nm as described in Methods. ^∗^*p* < 0.05*vs.* control. The SDH activity was measured spectrophotometrically at 600 nm as described in Methods. ^∗^*p* < 0.05*vs.* control.

Next, we investigated whether a decrease in glutamate/malate phosphorylation rates might be associated with the diminished activity of mitochondrial respiratory chain complexes. We revealed that even a short (20 min) ischemia/reperfusion period decreased complex I activity by 33% (Figure 2(a)). Longer (30 min, 40 min, and 60 min) ischemia/reperfusion periods further reinforced (by 56%, 62%, and 85%, *p* < 0.05) these changes.

As the succinate-dependent respiration rate was increased after 20-40 min of ischemia/reperfusion, next, we measured the activity of complex II+III in kidney mitochondria. There were no changes in complex II+III activity after 20 min of ischemia/reperfusion, but it slightly increased after 30 min and 40 min of ischemia/reperfusion (by 33% and 44%, respectively) (Figures 2(b), *p* > 0.05). After a longer (60 min) ischemia/reperfusion period, complex II+III activity decreased by 46% as compared with control (*p* < 0.05) and was supported by data of succinate respiration rate measurements. We also checked the activity of SDH and found that 20 min of ischemia/reperfusion had no effect on it (Figure 2(c)). When ischemia time was extended to 30 min and 40 min, the activity of SDH increased by 23% (*p* > 0.05) and by 65% (*p* < 0.05) as compared to control (nonischemic mitochondria), whereas after 60 min of ischemia/reperfusion, the SDH activity returned to the control level.

### 3.3. Ischemia/Reperfusion Causes Succinate Accumulation in Kidney Mitochondria

As in this study we found an increase in succinate respiration rate and in SDH activity after various periods of ischemia/reperfusion, next, we checked whether ischemia/reperfusion leads to accumulation of succinate in cytosolic and mitochondrial fractions. We found that after 30 min of ischemia/reperfusion, succinate concentration in the mitochondrial fraction increased by 15% as compared to control (*p* < 0.05) (Table 3), whereas the effect of 40 min ischemia/reperfusion on succinate content in mitochondria was similar to control (Table 3). Meanwhile, succinate content in the cytosolic fraction after 30 min ischemia/reperfusion increased more than 2 times as compared to control and continued to increase when ischemia time was prolonged to 40 min ischemia/reperfusion (*p* < 0.05) (Table 3).

### 3.4. Ischemia/Reperfusion Induces H_2_O_2_ Generation in Kidney Mitochondria

**Figure 3:**
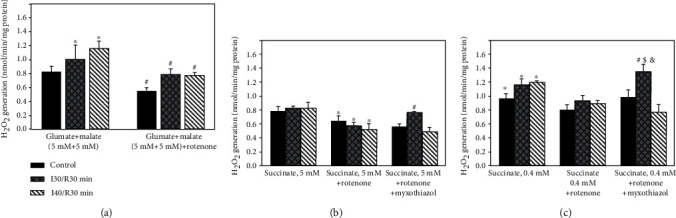
Effect of ischemia on H_2_O_2_ generation in kidney mitochondria with glutamate/malate (a) and succinate: saturating (b) and unsaturating (c) concentrations. The generation of H_2_O_2_ in kidney mitochondria was determined fluorimetrically as described in Methods (excitation at 544 nm, emission at 590 nm). Substrates: (a) glutamate (5 mM) + malate (5 mM); (b) succinate 5 mM; (c) succinate 0.4 mM. Respiratory chain complex inhibitors: rotenone (5 *μ*M) and myxothiazol (2 *μ*M). ^∗^*p* < 0.05 vs. succinate 5 mM group; ^#^*p* < 0.05 vs. control group; ^&^*p* < 0.05 vs. succinate 5 mM group; ^*λ*^*p* < 0.05 vs. control group; ^$^*p* < 0.05 vs. succinate 0.4 mM group; ^*ϕ*^*p* < 0.05 vs. succinate 5 mM, with rotenone group; ^§^*p* < 0.05 vs. succinate 5 mM, with rotenone and myxothiazol group; ^*ψ*^*p* < 0.05 vs. control group; ^*κ*^*p* < 0.05 vs. succinate 0.4 mM, with rotenone group; ^*α*^*p* < 0.05 vs. succinate 5 mM, with rotenone and myxothiazol group.

Our new finding was that succinate concentration in the mitochondrial as well as in cytosolic fractions increased during certain periods of ischemia; therefore, we tested the hypothesis of its relationship with the increased ROS production in mitochondria. We revealed that H_2_O_2_ generation rate with glutamate + malate increased by 23% and 41% (*p* < 0.05) after 30 and 40 min of ischemia/reperfusion, respectively (Figure 3). The addition of complex I inhibitor rotenone significantly decreased H_2_O_2_ generation in control and in ischemia/reperfusion affected mitochondria (Figure 3(a)).

The rate of H_2_O_2_ generation driven by saturating concentration of succinate (5 mM) in control mitochondria was 0.78 nmol/min mg protein, and this value was very similar to that induced by oxidation of glutamate + malate. H_2_O_2_ generation with 5 mM concentration of succinate did not change significantly when ischemia time was prolonged to 30 and 40 min (H_2_O_2_) (Figure 3(b)). Rotenone inhibited H_2_O_2_ generation in control and ischemia/reperfusion affected mitochondria by 18% and 37%, respectively (*p* < 0.05) (Figure 3(b)). When rotenone and complex III inhibitor myxothiazol were added together, H_2_O_2_ generation rate after 30 min ischemia/reperfusion increased by 36% (*p* < 0.05) as compared with control mitochondria and by 31% (*p* < 0.05) as compared with ROS generation when rotenone alone was added. When ischemia time was prolonged to 40 min, H_2_O_2_ generation decreased and returned to the control level.

Next, we found that H_2_O_2_ generation rate in control and in ischemia/reperfusion (30 min and 40 min) affected kidney mitochondria with subsaturating (0.4 mM) succinate concentration (Figure 3(c)) was higher by 23%, 40%, and 44%, respectively (*p* < 0.05), as compared to saturating (5 mM) succinate concentration. Thus, with a subsaturating succinate concentration, 30 min and 40 min ischemia/min reperfusion increased ROS generation by 35% (*p* > 0.05) and 44% (*p* < 0.05), respectively, as compared to the control group. The subsequent addition of rotenone significantly decreased H_2_O_2_ generation by 16% and 26%, in control and ischemia/reperfusion-affected mitochondria, respectively, as compared to the group without rotenone (Figure 3(c)). When rotenone and myxothiazol were added together, no effect on H_2_O_2_ generation was observed in control mitochondria, but it increased significantly after 30 min ischemia/reperfusion (Figure 3(c)). ROS generation after 30 min ischemia/reperfusion was 43% higher as compared to the control group, 47% higher as compared to the group when rotenone alone was added, and 77% higher as compared to the group with saturating succinate concentrations. The ischemia prolonged from 30 min to 40 min decreased H_2_O_2_ generation by 43% as compared to the shorter ischemia/reperfusion group.

### 3.5. Ischemia/Reperfusion Increases Lactate Dehydrogenase (LDH) Activity in Cytosolic Fraction

**Figure 4:**
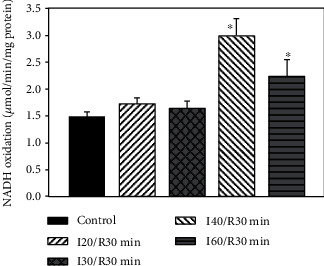
Effect of kidney ischemia on LDH activity in cytosolic fraction. LDH activity was measured as described in Methods. ^∗^*p* < 0.05*vs.* control.

LDH activity in the cytosolic fraction was measured as an indicator for tissue necrosis. In the cytosolic fraction of control kidneys, LDH activity was 1.48 ± 0.1 IU/min per mg protein. After 20 min and 30 min ischemia/30 min reperfusion, there was no significant change in LDH activity. LDH activity increased about 2 times after 40 min ischemia/min reperfusion and about 1.5 times after 60 min ischemia/min indicating severe damage of kidney tissue (Figure 4).

## 4. Discussion

Kidney ischemia/reperfusion injury is unavoidable during kidney surgery and depending on duration of ischemia which may affect kidney function with different severities of consequences. Many studies on effects of ischemia are performed *in vitro* using animal models, but it is always important to evaluate and compare the changes that occur in mitochondria *in vivo*. Furthermore, in this study, we determined if there is a connection among (a) duration of kidney ischemia and mitochondrial dysfunction and (b) succinate dehydrogenase activity, succinate accumulation, and ROS generation in mitochondria at low and saturating succinate concentrations.

In order to determinate changes that occur during *in vivo* ischemia/reperfusion injury, first, we measured the mitochondrial respiration rates with both glutamate/malate and succinate as mitochondrial substrates. Our results showed that mitochondrial phosphorylation rates with glutamate/malate decreased even after a short time (30 min) ischemia/reperfusion, and it was supported by the decreased activity of complex I. The prolongation of the ischemia period to 40 min and 60 min/reperfusion further reinforced mitochondrial damage (mitochondrial respiratory rates decreased by 86% and complex I activity by 85%). This study is in agreement with our results of the *in vitro* ischemia model [14] which already showed the disturbances in glutamate/malate respiration rates after 20 min and even more so after a longer (40 min and 60 min) ischemia period. Our *in vivo* study also revealed the damage of mitochondrial outer membrane and lack of cytochrome c after 30 min of ischemia/reperfusion (cytochrome c test), which increased drastically (two and four times) after 40 min and 60 min of ischemia/reperfusion respiring on succinate and supported our results *in vitro* [14]. However, our *in vivo* study revealed quite opposite results in terms of succinate dependent phosphorylation rates as compared to the *in vitro* model. We observed an increase in the succinate respiration rate after 20 min of ischemia/reperfusion, with a further increase after a prolonged (up to 40 min) period of ischemia. This result led us to test hypothesis and to measure complex II+III and SDH activity as well as succinate amount in mitochondrial and cytosolic fractions at various points of ischemia/reperfusion. Our quite new finding is that succinate concentration in the cytosolic fraction increased several times (2 and 4 times) after 30 min and 40 min of ischemia/reperfusion. Thus, it indicated accumulation of succinate in cytosol that occurred during the ischemia/reperfusion period. Moreover, our unpublished results revealed that after 30 min of ischemia alone, succinate concentration in the mitochondrial fraction increased 1.40 times and in the cytosolic fraction about 3 times. These results also indicate a damaging effect of ischemia on mitochondrial integrity. Remembering that succinate-dependent phosphorylation rates increased by 29%, 41% (*p* < 0.05), and 14% (*p* > 0.05) after 20 min, 30 min, and 40 min of ischemia/reperfusion, respectively, and the activity of complexes II+III and SDH increased by 20% and 65% after 30 min and 40 min of ischemia/reperfusion, (Figures 2(b) and 2(c)), it seems that the increase in the activity of these complexes can help to maintain the mitochondrial proton motive force during reperfusion, especially in the case when complex I activity decreased drastically, for instance, after 30 min and 40 min of ischemia/reperfusion (Figure 2(a)). Our data showed that prolonged ischemia (60 min ischemia/reperfusion) induced a significant decrease in complexes II+III and SDH activity (by 46% and 38%, respectively) (Figures 2(b) and 2(c)), as well as in succinate oxidation (by 80%) (Table 2). It is interesting to note that Gonzalez-Flecha and Boveris revealed that in kidney submitochondrial particles from the cortex, 30 min and 60 min ischemia had no effect on SDH activity and only a slight (about 15%) decrease of SDH activity after 10 min of reperfusion [15]. Moreover, our results on the increased activity of complexes II+III and SDH during certain periods of I/R were supported by data of the increased succinate accumulation after ischemia/reperfusion. Similar results were obtained by Andrienko et al.; they found that after 30 min of ischemia succinate concentration in a rat heart increased many times [16], and oxidation of succinate maintained the mitochondrial ATP synthesis. Therefore, to further confirm our hypothesis about the role of succinate in ROS production during ischemia/reperfusion injury, we investigated the relationship between the activity of SDH, succinate accumulation, and production of ROS; it is generally acknowledged that ischemia/reperfusion-induced mitochondrial dysfunction might be associated with overproduction of ROS, generated by several mechanisms. It was shown by others that complexes I and II can produce ROS into the matrix and complex III can produce ROS on both sides of the mitochondrial inner membrane [17]. Recently, it was shown that ROS generation driven by complex II is especially important under pathological conditions when different parts of the respiratory chain can be inhibited [18], and therefore, succinate accumulation (which arises in ischemia from reversal of succinate dehydrogenase) can be associated with mitochondrial ROS production during reperfusion [19, 20]. This conclusion was supported by our present results. Thus, we revealed that accumulation of succinate in the cytosol after ischemia/reperfusion correlates with the increased SDH activity and H_2_O_2_ generation (Figures 2(b), 2(c), and 3(c)). H_2_O_2_ generation mediated by SDH (after addition of rotenone and myxothiazol) was the most noticeable with physiological succinate concentration after 30 min of ischemia/reperfusion (Figure 3(c)). It is important to note that for measurement of ROS generation during ischemia/reperfusion most of studies used only saturating concentrations of succinate. This is not sufficient for understanding the changes that occur under *in vivo* conditions. In the present study, we used physiological (0.4 mM) succinate concentrations in order to detect changes that occur under *in vivo* conditions in ischemia/reperfusion and revealed the role of SDH in ROS generation during kidney ischemia/reperfusion. Chouchani et al. [7] observed the accumulation of succinate after ischemia and noticed that ischemic succinate accumulation was responsible for mitochondrial ROS production during reperfusion through a reverse electron transport at mitochondrial complex I [7]. Thus, our results indicate that ROS generation by reverse electron transport driven by complex I in kidney mitochondria is only one of several pathways. SDH, as was shown in our study, is also involved in ROS production, especially at physiological succinate concentrations (Figure 3(c)), and this is in line with Quinlan et al.'s observation that ROS production at complex II in muscle mitochondria occurs at low concentration of succinate when the succinate-binding site is not occupied by substrate and that ROS generation is inhibited by excess of succinate [8].

Furthermore, ischemia-induced accumulation of succinate in the cytosol and ROS generation (Table 3, Figures 3(b) and 3(c)) mediates inflammatory response leading to cell death. The 2 and 1.5 times increased LDH activity after 40 min and 60 min of ischemia/reperfusion, an indicator for necrosis, was observed in our study and was in line with other studies (Figure 4) [14, 21, 22]. It is important to know that strict regulation of ROS levels is crucial for cellular life. In the cell, H_2_O_2_ plays the role of a second messenger and is involved in the control of gene expression and contribute to the control of cell proliferation and differentiation [23]. On the other hand, a moderate increase of ROS contributes to several pathologic conditions, among which are ischemia-reperfusion, inflammation, tumor promotion, and progression. So the development of therapeutic strategies which modulate mitochondrial and cellular ROS levels can be used for disease prevention or treatment [23].

In summary, our results point out that (1) mitochondrial disturbances can occur even after 30 min of kidney ischemia/reperfusion *in vivo* and increase progressively with the prolonged time of ischemia; (2) accumulation of succinate in cytosol after ischemia/reperfusion correlated with increased H_2_O_2_ generation mediated by complex II, which was most noticeable with physiological succinate concentrations; and (3) ischemia/reperfusion induced cell necrosis, indicated by the changes in LDH activity.

## 5. Conclusion

Thus, our new findings on the accumulation of succinate in cytosol and changes in SDH activity during kidney ischemia/reperfusion may be important for energy production after reperfusion, when complex I activity is suppressed. On the other hand, an increased activity of succinate dehydrogenase is associated with the increased ROS generation, especially with physiological succinate concentrations. All these observations play an important role in understanding the mechanisms which occur in the early phase of ischemia/reperfusion injury *in vivo* and may provide new ideas for novel therapeutic approaches or injury prevention; therefore, more detailed studies are necessary in the future.

## Figures and Tables

**Table 1:** Effects of ischemia/reperfusion on kidney mitochondrial phosphorylation rates with glutamate/malate as substrates.

	*V*_0__(Glu)_	*V*_3__(Glu)_	*V*_3+cyt c_ _(Glu)_	*V*_ATR_ _(+cyt c)_	RCI _(Glu)_	cyt c effect
Control	67 ± 7	210 ± 17	181 ± 18	75 ± 6	3.34 ± 0.27	0.87 ± 0.04
I20/R30 min	67 ± 4	205 ± 19	192 ± 17	71 ± 6	3.08 ± 0.34	0.94 ± 0.03
I30/R30 min	63 ± 9	132 ± 9^∗^	127 ± 11^∗^	56 ± 7	2.39 ± 0.48	0.96 ± 0.04
I40/R30 min	31 ± 2^∗^	50 ± 5^∗^	63 ± 4^∗^	32 ± 1^∗^	1.64 ± 0.15^∗^	1.30 ± 0.12^∗^
I60/R30 min	34 ± 4^∗^	30 ± 4^∗^	38 ± 6^∗^	26 ± 4^∗^	0.89 ± 0.03^∗^	1.26 ± 0.03^∗^

Mitochondrial respiration rate was measured as described in Methods using 5 mM glutamate plus 5 mM malate as substrates. *V*_0_: routine respiration rate in the presence of 0.5 mg/mL of mitochondria and substrates; *V*_3_: state 3 respiration rate in the presence of 1 mM ADP; *V*_3+cyt c_: state 3 respiration rate in the presence of 32 *μ*M cytochrome c; *V*_ATR (+cyt c)_: respiration rate in the presence of atractyloside 0.12 mM and 32 *μ*M cytochrome c. Mitochondrial respiratory control index (RCI), i.e., the ratio of oxygen uptake rate in state 3 to routine respiration rate (RCI = *V*_3_/*V*_0_). Cytochrome c effect (cyt c effect _(Succ)_), i.e., the ratio of oxygen uptake rate in state 3 (in the presence of cytochrome c) to state 3 respiration rate (cyt c effect = *V*_3+cyt c_/*V*_3_). ^∗^*p* < 0.05*vs.* control.

**Table 2:** Effects of ischemia/reperfusion on kidney mitochondrial phosphorylation rates with succinate as substrate.

	*V*_0__(Succ)_	*V*_3__(Succ)_	*V*_3+cyt c_ _(Succ)_	*V*_ATR_ _(+cyt c)_	RCI _(Glu)_	cyt c effect
Control	153 ± 20	353 ± 33	382 ± 34	236 ± 23	2.49 ± 0.27	1.08 ± 0.03
I20/R30 min	229 ± 21	454 ± 15^∗^	506 ± 21^∗^	286 ± 11	2.04 ± 0.20	1.11 ± 0.02
I30/R30 min	206 ± 17	499 ± 58^∗^	633 ± 85^∗^	334 ± 46	2.39 ± 0.12	1.27 ± 0.07^∗^
I40/R30 min	262 ± 35^∗^	405 ± 80	677 ± 97^∗^	467 ± 62^∗^	1.47 ± 0.15^∗^	1.86 ± 0.23^∗^
I60/R30 min	71 ± 15^∗^	75 ± 17^∗^	266 ± 26^∗^	235 ± 17^∗^	1.05 ± 0.04^∗^	3.80 ± 0.46^∗^

Mitochondrial respiration rate was measured as described in Methods using 15 mM succinate (+2 mM amytal) as substrate. *V*_0_: routine respiration rate in the presence of 0.5 mg/mL of mitochondria and substrates; *V*_3_: state 3 respiration rate in the presence of 1 mM ADP; *V*_3+cyt c_: state 3 respiration rate in the presence of 32 *μ*M cytochrome c; *V*_ATR (+cyt c)_: respiration rate in the presence of atractyloside 0.12 mM and 32 *μ*M cytochrome c. Mitochondrial respiratory control index (RCI), i.e., the ratio of oxygen uptake rate in state 3 to routine respiration rate (RCI = *V*_3_/*V*_0_). ^∗^*p* < 0.05*vs.* control.

**Table 3:** Effect of ischemia/reperfusion on succinate content in the kidney (*μ*g/g tissue).

	Succinate content in mitochondria	Succinate content in cytosol
Control group	112.3 ± 4.4	8.1 ± 1.4
I30/R30 min	129.1 ± 3.0^∗^	19.2 ± 2.0^#^
I40/R30 min	114.9 ± 2.2	35.3 ± 1.4^#^

Succinate concentration in mitochondria and cytosolic fractions was measured as described in Measurement of H_2_O_2_ Generation in Kidney Mitochondria. ^∗^*p* < 0.05*vs.* control; ^#^*p* < 0.05*vs.* control.

## Data Availability

The datasets used and analyzed during the current study are available from the corresponding author on reasonable request.

## Abbreviations

I/R: Ischemia/reperfusion
LDH: Lactate dehydrogenase
RCI: Respiratory control index
SDH: Succinate dehydrogenase
ROS: Reactive oxygen species.
